# Improving the efficiency of cost-effectiveness analysis to inform policy decisions in the real world: lessons from the Pharmacoeconomics Research Unit at Cancer Care Ontario

**DOI:** 10.1186/1472-6963-14-S2-O39

**Published:** 2014-07-07

**Authors:** Jeffrey S Hoch

**Affiliations:** 1 Institute of Health Policy, Management and Evaluation, University of Toronto, Canada

## 

There are important challenges in the application of cost-effectiveness analysis results in the real world that highlight the great divide between academic research and practical application. The difficulty is magnified in cancer because of the intense emotions it raises and their influence on decision making, impacting treatment funding decisions. Nevertheless, the potential for cost-effectiveness analysis to inform policy decisions is also great.

In 2007, Cancer Care Ontario (CCO) established Canada’s first in-house Pharmacoeconomics Research Unit comprised of independent researchers. This presentation reviews the initial five years of the Pharmacoeconomics Research Unit at CCO. The purpose is to share lessons and point out directions for future research in cost-effectiveness analysis aimed at informing decision makers in the real world.

To enrich the evidence base, we must continue to develop and apply new methods of analyzing data and displaying information. We must also face the reality that the purpose of our role may be to promote goals related to process rather than outcome, suggesting that getting the question of interest right may be more important for researchers than correctly solving the wrong problem. Creating the best estimate of cost-effectiveness is not knowledge for knowledge’s sake; this type of information is the foundation of accountability and sustainability. There is great potential for methods from health economics to provide useful information; it is possible for the results of our analysis to be understood and used by policy makers and other decision makers in the real world. Experiences at the Pharmacoeconomics Research Unit have illustrated this potential.

